# The acute transcriptional responses to dietary methionine restriction are triggered by inhibition of ternary complex formation and linked to Erk1/2, mTOR, and ATF4

**DOI:** 10.1038/s41598-021-83380-0

**Published:** 2021-02-12

**Authors:** Kirsten P. Stone, Sujoy Ghosh, Jean Paul Kovalik, Manda Orgeron, Desiree Wanders, Landon C. Sims, Thomas W. Gettys

**Affiliations:** 1 Laboratory of Nutrient Sensing and Adipocyte Signaling, Pennington Biomedical Research Center, 6400 Perkins Road, Baton Rouge, LA 70808 USA; 2 Laboratory of Computational Biology, Pennington Biomedical Research Center, Baton Rouge, LA USA; 3 Program in Cardiovascular and Metabolic Disorders and Center for Computational Biology, Duke-NUS Medical School, Singapore, Singapore; 4 Department of Nutrition, Georgia State University, Atlanta, GA USA

**Keywords:** Cell biology, Physiology

## Abstract

The initial sensing of dietary methionine restriction (MR) occurs in the liver where it activates an integrated stress response (ISR) that quickly reduces methionine utilization. The ISR program is regulated in part by ATF4, but ATF4’s prototypical upstream regulator, eIF2α, is not acutely activated by MR. Bioinformatic analysis of RNAseq and metabolomics data from liver samples harvested 3 h and 6 h after initiating MR shows that general translation is inhibited at the level of ternary complex formation by an acute 50% reduction of hepatic methionine that limits formation of initiator methionine tRNA. The resulting ISR is induced by selective expression of ATF4 target genes that mediate adaptation to reduced methionine intake and return hepatic methionine to control levels within 4 days of starting the diet. Complementary in vitro experiments in HepG2 cells after knockdown of ATF4, or inhibition of mTOR or Erk1/2 support the conclusion that the early induction of genes by MR is partially dependent on ATF4 and regulated by both mTOR and Erk1/2. Taken together, these data show that initiation of dietary MR induces an mTOR- and Erk1/2-dependent stress response that is linked to ATF4 by the sharp, initial drop in hepatic methionine and resulting repression of translation pre-initiation.

## Introduction

Initiation of dietary methionine restriction (MR) produces a series of physiological responses linked to transcriptional programs that are regulated by hepatic sensing of reduced methionine^1–4^. An important advance in understanding how dietary MR produces its metabolic phenotype came from the observation that hepatic fibroblast growth factor 21 (FGF21) was a component of an Integrated Stress Response (ISR) activated within hours of introduction of the MR diet^5–7^. Subsequent loss of function studies showed that the ability of dietary MR to increase energy expenditure (EE), reduce fat deposition, and remodel white adipose tissue was dependent on FGF21^6^. Dietary MR also reduces the capacity of the liver to synthesize and export fat through transcriptional mechanisms^2,6^. Collectively, these responses allow immediate adaptation to the shortage of methionine while improving the metabolic health of the animal in the weeks following introduction of the MR diet^7,8^.

The nutrient sensing systems that detect methionine restriction and initiate the ISR have been the subject of intense study, but the chronological progression of adaptive responses that occur after initiation of MR have made their identification difficult. For example, the accepted view is that general control nonderepressible 2 (GCN2) senses decreased tRNA charging after a reduction in hepatic amino acids and initiates the ISR through increased phosphorylation of eukaryotic initiation factor 2 alpha (eIF2α)^9–16^. Within this scheme, the activated eIF2α then slows overall translation while also directing preferential translation of select mRNAs including activating transcription factor 4 (ATF4). Newly synthesized ATF4 then coordinates key elements of the ISR by binding to promoters of target genes. One of ATF4’s key targets is hepatic FGF21, which acts as a powerful metabolic regulator in the context of glucose homeostasis, lipid metabolism, and energy balance^17–23^. An alternative pathway to ATF4 activation has been suggested by more recent work showing that MR can promote ATF4 activity independently of increased eIF2α phosphorylation^24^. Collectively, these findings support the existence of additional signaling mechanisms that function as redundant nutrient sensing systems.

In the present work we have focused on the responses that occur within hours after introduction of the MR diet. In this time frame, initiation of translation requires formation of ternary complexes consisting of GTP, initiator methionine tRNA (Met-tRNA) and eIF2α^25,26^. GCN2-dependent phosphorylation of eIF2α normally inhibits formation of this complex, but recent work shows that ATF4 target gene expression is also modulated by MR through a mechanism independent of both GCN2 and eIF2α^27,28^. This mechanism primarily impairs start site recognition due to insufficient Met-tRNA^28,29^. It is possible that the acute responses to MR involve a similar inhibition of ternary complex formation due to the precipitous decrease in methionine. Additional eIF2α-independent pathways include mechanistic target of rapamycin (mTOR) that induces ATF4 target genes^30,31^ through mTOR-dependent stabilization of ATF4 mRNA^30^. However, a role for mTOR as a sensor and transcriptional mediator of dietary MR has not been previously established. Using a combination of RNAseq and metabolomics analysis of mouse liver samples taken 3 h and 6 h after introduction of dietary MR, in conjunction with in vitro analysis of methionine-restricted HepG2 cells, our data suggest that the rapid initial decrease in hepatic methionine decreases ternary complex formation, slows overall translation, and induces an ISR that is modulated by mTOR, Erk1/2, and ATF4.

## Results

**Figure 1:**
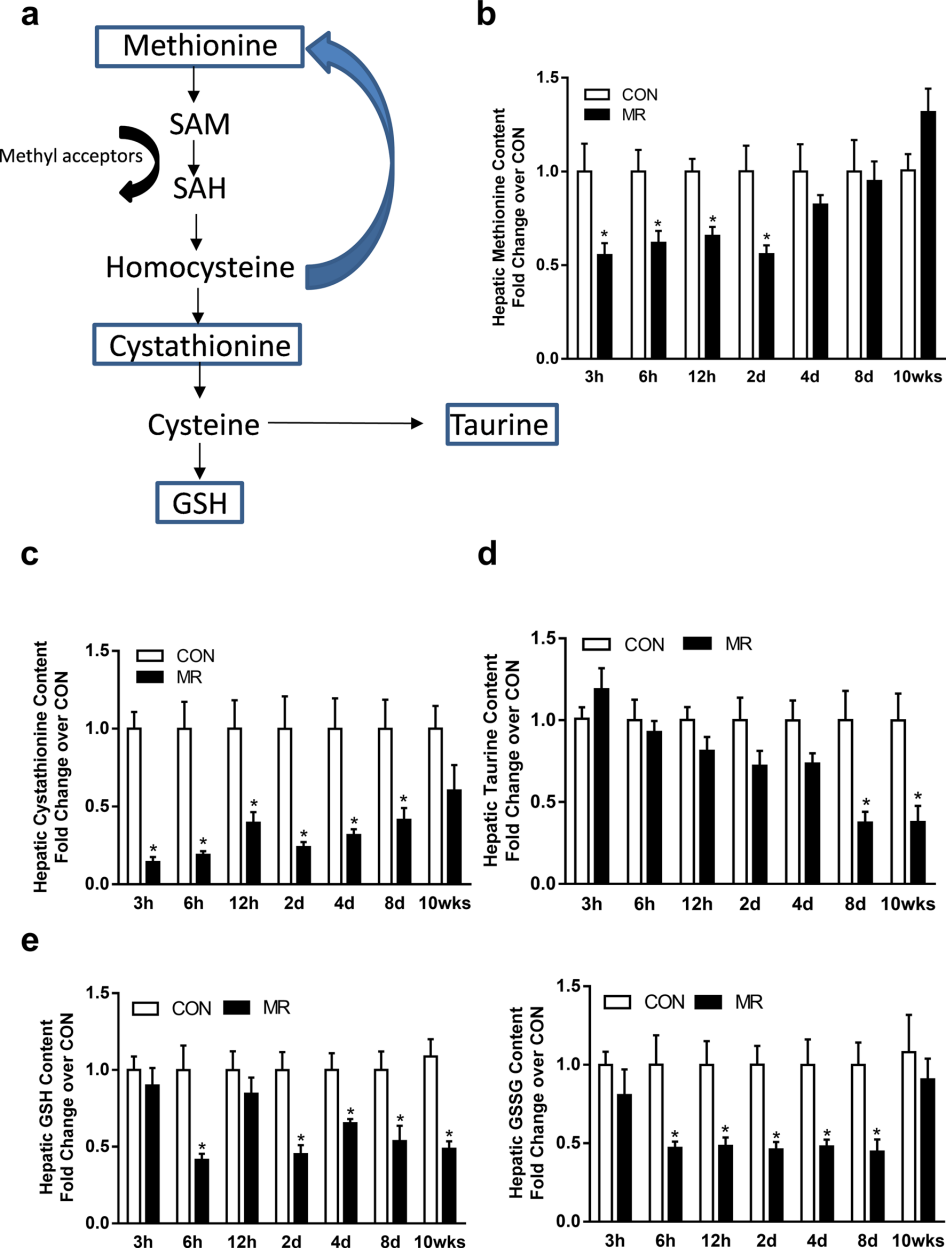
Schematic presentation of methionine metabolism (**a**). Hepatic methionine (**b**), cystathionine (**c**), taurine (**d**), GSH and GSSG (**e**) were measured using metabolomics analysis of mice exposed to Con or MR for 3 h, 6 h, 12 h, 2 days, 4 days, 8 days, and 10 weeks. Data were obtained from n = 8/group and compared between Con and MR for each time point using t-test *p < 0.05. Data are presented as fold induction from Con at each time point.

The MR diet is devoid of cysteine and contains fivefold less methionine than the Con diet, so a key goal of *Experiment 1* was to assess acute, MR-induced changes in key metabolites of methionine metabolism in the liver (Fig. 1a). The MR diet reduced hepatic methionine by 50% within 3 h of starting the diet (Fig. 1b). Methionine levels remained significantly lower in the MR group for the first 2 days, but by day 4, were restored to Con levels where they remained thereafter (Fig. 1b). The formation of cystathionine from homocysteine is irreversible and the first reaction of the trans-sulfuration pathway (Fig. 1a). Within 3 h of starting the MR diet, cystathionine levels were reduced by > 80% (Fig. 1c) without a change in expression of Cystathionine-β-synthase and Cystathionine gamma-lyase (RNAseq data). The magnitude of the cystathionine reduction decreased over the following 8 days, but levels in the MR group remained significantly lower than in Con mice over this period (Fig. 1c). Cysteine is formed from cystathionine and serves as a precursor for both taurine and glutathione synthesis in the liver (Fig. 1a). Although taurine is present at high levels in the bloodstream and synthesized in multiple tissues, the majority of whole body biosynthesis occurs in the liver. Interestingly, despite the rapid reduction in hepatic glutathione in the MR group (Fig. 1e), taurine levels were unaffected by the MR diet for the first 4 days, and not until day 8 did a significant reduction in taurine occur in the MR group (Fig. 1d). Taurine is absent from both diets but it has a long biological half-life in mice^40^. The combination of synthesis and renal reabsorption^41^ are the primary determinants of taurine levels in the body. However, it is unclear how hepatic levels of taurine are maintained at control levels during the early stages of MR while other metabolites of methionine metabolism are acutely affected. For example, the oxidized (e.g., GSSG) and reduced (e.g., GSH) forms of glutathione were both reduced by 50% in the MR group within the first 6 h (Fig. 1e), and the levels of both forms remained 50% lower than Con for the initial 8 days. GSH was also 50% lower in the MR group after 10 weeks on the MR diet (Fig. 1e).

**Figure 2:**
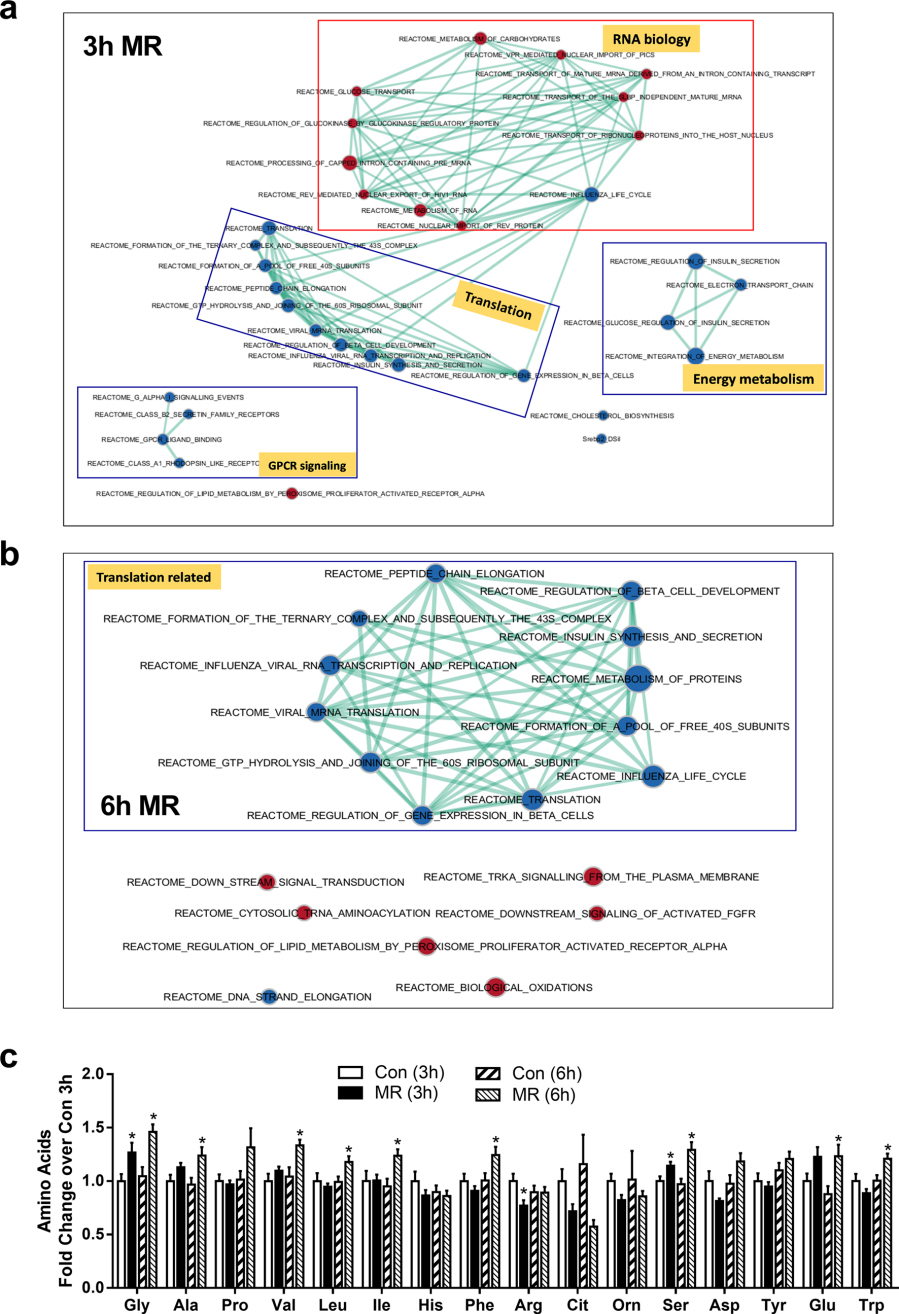
GSEA from livers of mice exposed to Con or MR for either 3 h (**a**) or 6 h (**b**). GSEA was derived from RNAseq of each 6 liver samples from mice fed either Con or MR for 3 h and 6 h and analyzed as described in Methods. Data was analyzed via EnrichmentMap (version 2.2.1, apps.cytoscape.org/enrichmentmap) and visualized in Cytoscape (version 3.8.0, www.cytoscape.org). (**c**) Hepatic amino acids of mice exposed to Con or MR for 3 h and 6 h were quantified using metabolomics analysis. Data were obtained from n = 8/group and compared between Con and MR for each time point using unpaired t-test *p < 0.05. Data are presented as fold induction from Con at 3 h.

**Figure 3:**
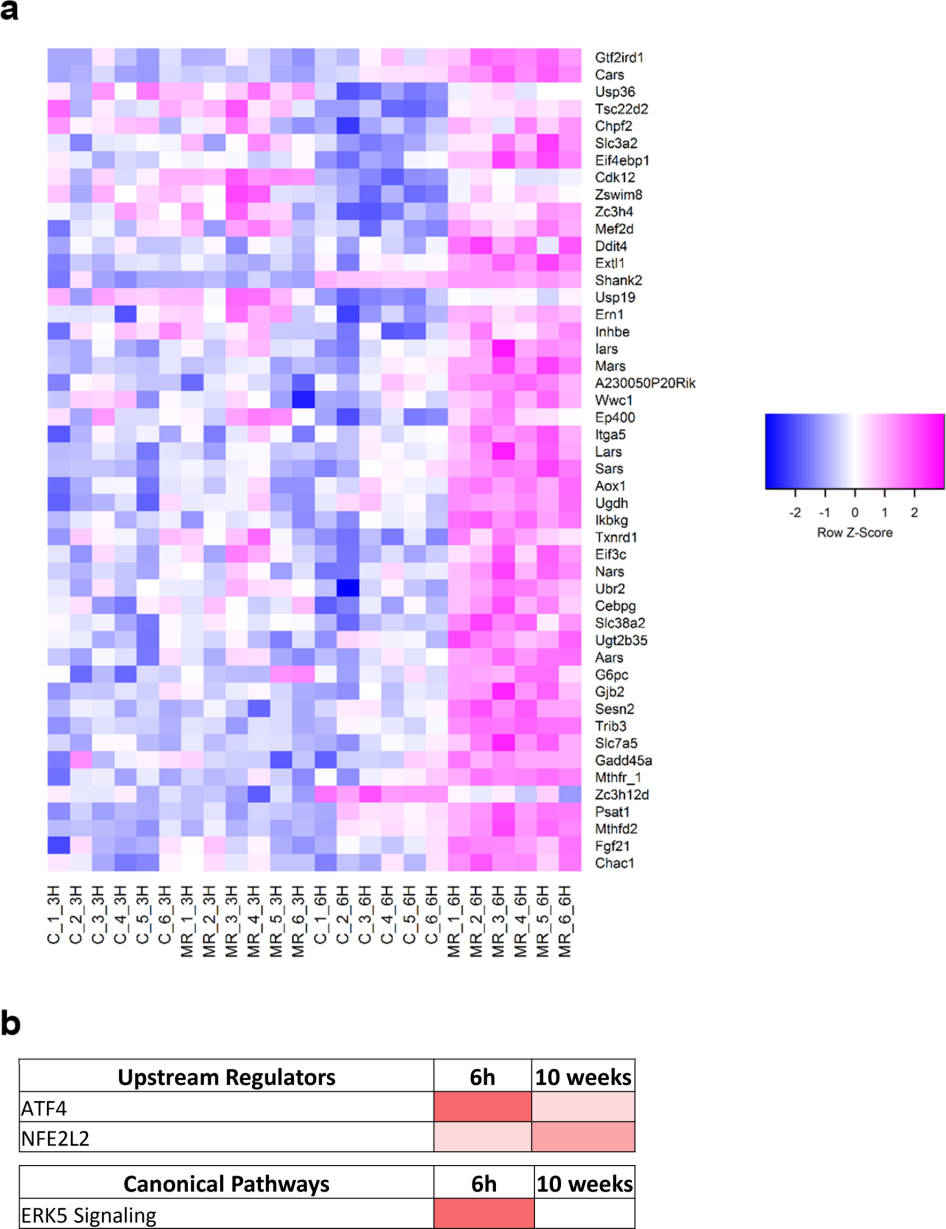

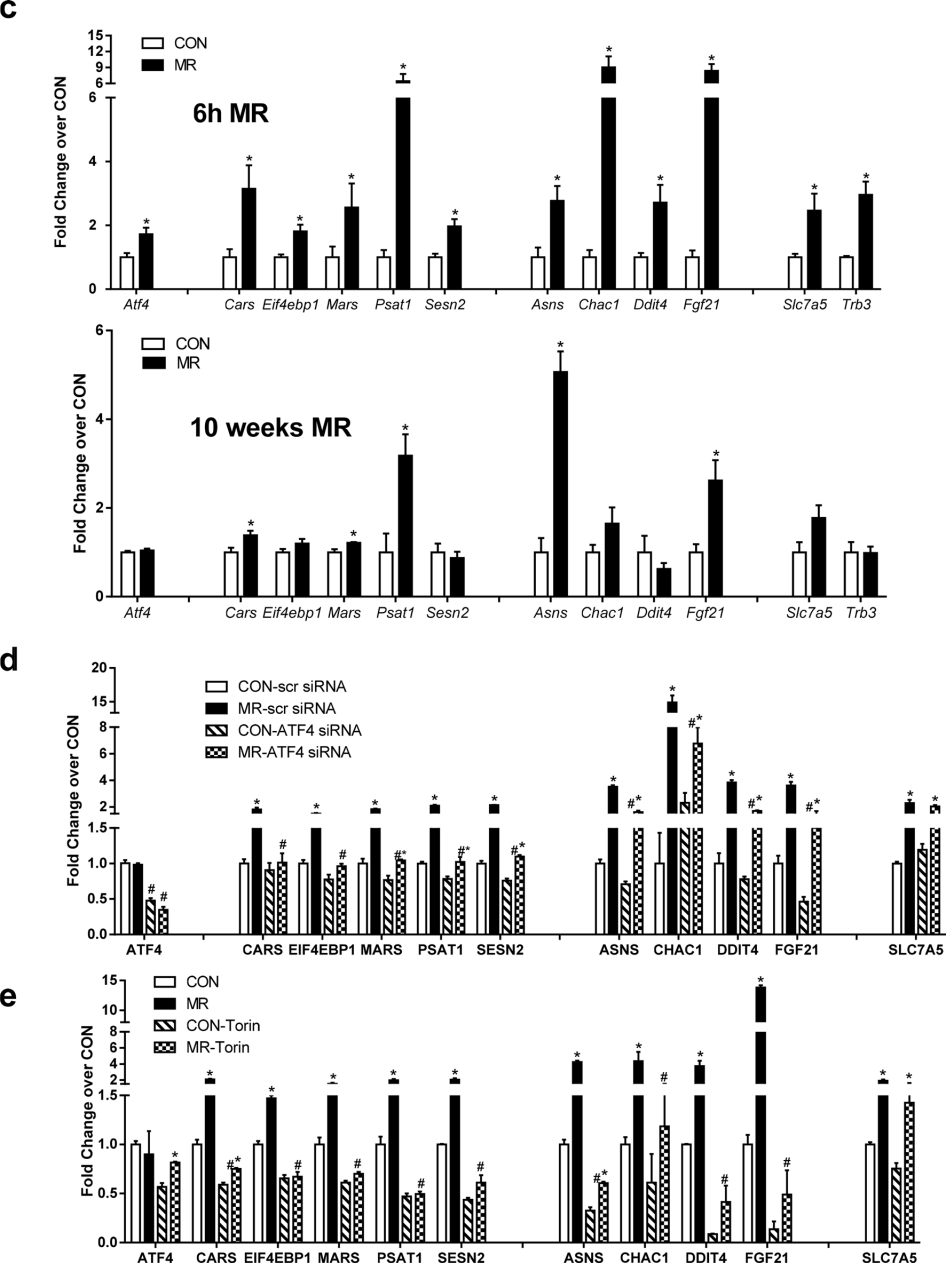
Heatmap of liver genes induced after 6 h on MR (**a**). Genes significantly different in MR versus Con at 6 h (FDR <  = 0.001) are presented based on the magnitude of fold changes. The expression of these genes was measured using RNAseq and is shown for each of the 3 h and 6 h samples. Heatmaps were constructed via the gplots package in R (version 2.2.0, https://cran.r-project.org/web/packages/gplots/index.html) (**b**) Comparison of liver samples from mice fed Con or MR for either 6 h or 10 weeks. Upstream regulators and canonical pathways were identified by IPA analysis and are shown according to z-scores (n = 6/group; activating in red). (**c**) Gene expression in mouse livers after either 6 h (upper panel) or 10 weeks (lower panel) on Con or MR was determined by qPCR (n = 8/group). (**d**) HepG2 cells were transfected with either scrambled or ATF4 siRNA and gene expression was measured after cultivation for 6 h in either Con or MR media. (**e**) HepG2 cells were treated with either vehicle (DMSO) or Torin (200 nM) and then exposed to either Con or MR media for 6 h. Data for HepG2 cells were obtained from at least two independent experiments with duplicates, and fold induction was averaged. Data were compared between Con and MR and between MR and MR with ATF4siRNA (**D**) or MR with Torin (**E**) for each gene using unpaired t-test and are presented as fold induction from Con. * Means annotated with an asterisk differ from Con at p < 0.05, while means differing between MR without and MR with ATF4siRNA (**D**) or Torin (**E**) at p < 0.05 are annotated with the pound symbol ^#^.

We previously showed that hepatic FGF21 expression and release were increased within hours of starting the MR diet^5,27^, but the sensing mechanism linking reduced methionine to transcriptional activation of FGF21 is unknown. Based on present findings that the acute effects on methionine metabolism were evident within 3 h of starting the MR diet, we used RNAseq and bioinformatic analysis at the corresponding time points to interrogate the transcriptional responses in livers from mice on the respective diets. To explore the systems biology of acute transcriptional responses to MR, differentially expressed genes were screened against annotated databases to identify gene set enrichment within biological processes and detect activation or inhibition of canonical pathways. Gene set enrichment analysis at the 3 h time point showed that RNA biology-dependent pathways were activated, whereas overall translation, energy metabolism, and inhibitory G protein-coupled receptor (GPCR) signaling were suppressed (Fig. 2a). RNA biology includes transport and metabolism of carbohydrates, glycolysis, processing of pre-mRNA, and nuclear import and export events (Table 1). In contrast, both the electron transport chain and general translation were inhibited at 3 h (Table 1). Suppression of general translation was also the major enriched pathway after 6 h MR (Fig. 2b, Table 1), and this finding was consistent with the predicted shortage of Met-tRNA and a corresponding suppression of ternary complex formation at these early time points (Fig. 1b, Table 1). Reactome pathways upregulated after both 3 and 6 h MR include tRNA aminoacylation, downstream signaling of activated Fibroblast Growth Factor Receptor, carbohydrate metabolism, and regulation of lipid metabolism by peroxisome proliferator activated receptor alpha (Table 1, Fig. 2b). Metabolomics analysis of hepatic amino acids complemented the gene set enrichment data by showing that although methionine concentrations were reduced by 50% after 3 h of MR (Fig. 1b), Glycine (Gly) concentrations were actually increased at this time point. In addition, Gly, Leucine (Leu), Ileucine (Ile), Phenylalanine (Phe), Glutamic acid (Glu), and Tryptophan (Trp) concentrations were all significantly increased by MR at the 6 h time point (Fig. 2c). As shown in Table 2, Alanine (Ala), Aspartic acid (Asp), Cysteine (Cys), Serine (Ser), Glutamine (Gln), Proline (Pro), and Asparagine (Asn) were not provided in the diet. Analysis of differential gene expression identified a significant number of hepatic genes previously associated with MR-induced effects on the ISR through activation of ATF4 and Nuclear factor erythroid 2-related factor 2 (NFE2L2)^27^. Figure 3a illustrates that these genes were upregulated at 6 h but minimally affected at 3 h. Upstream regulator analysis of the 6 h time point, in comparison to the 10 week time point, was used to identify acutely activated transcription factors that may or may not have been chronically affected by MR (Fig. 3b). This algorithm predicts changes in transcription factor activity based on observed patterns of change in gene expression. The analysis identified changes consistent with an ISR mediated in part through strong ATF4 activation at the 6 h time point that persisted at 10 weeks, and a significant activation of NFE2L2 at both time points (Fig. 3b). For example, a detailed analysis of known ATF4 target genes in mouse liver showed that expression of ATF4 itself, Glutathione-specific gamma-glutamylcyclotransferase (*Chac1*), DNA Damage Inducible Transcript 4 (*Ddit4*), Eukaryotic initiation factor 4e binding protein 1 (*Eif4ebp1*), Sestrin 2 (*Sesn2*), Solute Carrier Family 7 Member 5 (*Slc7a5*), and Tribbles homolog 3 (*Trb3*) were increased after 6 h MR but to a lesser extent after 10 weeks on the MR diet (Fig. 3c). The MR-induced increases in other components of this gene set (cysteinyl-tRNA synthetase, *Cars*; methionyl -tRNA synthetase, *Mars*; Phosphoserine Aminotransferase, *Psat1*; *FGF21*) were also significantly lower after 10 weeks of MR than after 6 h (Fig. 3c). In contrast, the prototypical ATF4 target gene, Asparagine Synthetase (*ASNS*) was upregulated to a greater extent after chronic consumption of the MR diet (Fig. 3c). Collectively, the findings suggest that ATF4 is not the sole transcriptional mediator of MR and that the diet is producing additional regulatory input to genes of the ISR^27^.

**Table 1:** Significantly up/down-regulated Reactome pathways in MR at 3 h, 6 h.

Reactome pathways	DnMR MRvsCon 3 h	DnMR MRvsCon 6 h	UpMR MRvsCon 3 h	UpMR MRvsCon 6 h
Biological oxidations				2.396715
Cholesterol biosynthesis	**−** 3.4078822			
Class A1 rhodopsin like receptors	**−** 2.326375			
Class B2 secretin family receptors	**−** 2.7220101			
Cytochrome P450 arranged by substrate type			2.046408	
Cytosolic TRNA aminoacylation			**2.4205055**	**2.5490904**
Dna strand elongation		**−** 2.992151		
Down stream signal transduction				2.1664991
Downstream events in GPCR signaling	**−** 1.818265			
Downstream signaling of activated FGFR			**1.955468**	**2.2847009**
Electron transport chain	**−** **3.843558**	**−** **2.3826458**		
Extension of telomeres		**−** 2.291407		
Formation of a pool of free 40s subunits	**−** **4.3410554**	**−** **4.1107283**		
Formation of the ternary complex and subsequently the 43s complex	**−** **2.4353325**	**−** **2.7291327**		
G alpha I signalling events	**−** 2.689316			
G alpha S signalling events	**−** 1.9093605			
Generic transcription pathway				2.0528708
Glucose metabolism			2.250311	
Glucose regulation of insulin secretion	**−** 2.473282			
Glucose transport			2.9154043	
GPCR ligand binding	**−** 3.0853083			
GTP hydrolysis and joining of the 60s ribosomal subunit	**−** **3.8503613**	**−** **3.6869833**		
Immunoregulatory interactions between a lymphoid and a non lymphoid cell		**−** 2.164804		
Influenza life cycle	**−** **3.3765147**	**−** **2.6112316**		
Influenza viral RNA transcription and replication	**−** **4.57597**	**−** **3.2909338**		
Insulin synthesis and secretion	**−** **4.889476**	**−** **4.9580593**		
Integration of energy metabolism	**−** 2.2607431			
IRS related events				2.1780689
Lagging strand synthesis		**−** 2.2954504		
Metabolism of carbohydrates			**3.0271425**	**2.0608919**
Metabolism of lipids and lipoproteins				2.4643822
Metabolism of proteins	**−** 2.4172044	**−** 2.664845		
Metabolism of RNA			2.5664449	
Muscle contraction	**−** 2.0547333			
NEP NS2 interacts with the cellular export machinery			2.4746692	
Nuclear import of Rev protein			2.384731	
Peptide chain elongation	**−** **4.97241**	**−** **4.264338**		
Peptide ligand binding receptors	**−** 1.811924			
Phase 1 functionalization of compounds				1.983418
PI3K AKT signaling				2.1251023
Processing of capped intron containing pre MRNA			2.570463	
Regulation of beta cell development	**−** **4.3684816**	**−** **3.691408**		
Regulation of gene expression in beta cells	**−** **4.5289383**	**−** **3.8040934**		
Regulation of glucokinase by glucokinase regulatory protein			2.8642175	
Regulation of insulin secretion	**−** 2.7360766			
Regulation of lipid metabolism by peroxisome proliferator activated receptor alpha			**2.6033673**	**2.2035155**
Rev mediated nuclear export of HIV1 RNA			2.5740607	
Signaling by EGFR				2.0452437
Signalling by NGF				2.2262647
Smooth muscle contraction	**−** 1.9416506			
Snrnp assembly			2.6126812	
Steroid metabolism	**−** 1.9754717			
Translation	**−** **3.1805887**	**−** **3.5010388**		
Translation initiation complex formation	**−** **1.8502407**	**−** **2.1808248**		
Transmembrane transport of small molecules			2.0095303	
Transport of mature MRNA derived from an intron containing transcript			2.6713839	
Transport of ribonucleoproteins into the host nucleus			2.5720353	
Transport of the SLBP independent mature MRNA			2.743715	
TRKA signalling from the plasma membrane				2.338559
TRNA aminoacylation			**2.127703**	**1.9886005**
Viral MRNA translation	**−** **4.902635**	**−** **4.0746527**		
VPR mediated nuclear import of pics			2.750522	

Reactome pathways that are significantly (FDR < 5%) up- or down-regulated in MR at 3 h and 6 h were identified via GSEA pre-ranked. Pathways identified in > 1 condition are highlighted. Several pathways share genes among them—these are identified via Enrichment Map analysis (Fig. 2A,B).

**Table 2:** Dietary amino acid composition.

	Con (%)	MR (%)
Glutamic acid	2.7	3.39
Proline (Pro)	0	0
Leucine (Leu)*	1.11	1.11
Lysine (Lys)*	1.8	1.8
Valine (Val)*	0.82	0.82
Aspartate (Asp)	0	0
Serine (Ser)	0	0
Tyrosine (Tyr)	0	0
Isoleucine (Ile)*	0.82	0.82
Phenylalanine (Phe)*	1.16	1.16
Threonine (Thr)*	0.82	0.82
Arginine (Arg)	1.12	1.12
Histidine (His)	0.33	0.33
Alanine (Ala)	0	0
Methionine (Met)*	0.86	0.17
Glycine (Gly)	2.33	2.33
Tryptophan (Trp)*	0.18	0.18
Cystine (Cys)	0	0

*Indicates essential amino acids.

To further clarify the role of ATF4 as a mediator of the acute ISR to MR, we used siRNA to knock down ATF4 in HepG2 cells prior to in vitro methionine restriction. ATF4 siRNA produced a 50% decrease in ATF4 mRNA that was sufficient to blunt but not block the MR-induced increases of some ATF4 target genes (e.g., ASNS, CHAC1, DDIT4, FGF21), while fully inhibiting MR-induced increases of other ATF4 target genes (e.g., CARS, MARS, EIF4EBP1, PSAT1, and SESN2) (Fig. 3d). The knockdown of ATF4 also appeared to modestly reduce basal expression of approximately 6 of the 10 genes in control cells (Fig. 3d). Induction of the hepatic ATF4 target genes (*Cars, Mars, Eif4ebp1, Psat1, Sesn2*) by MR is not dependent on Gcn2 as it is similar between wild type and Gcn2^−/−^ mice fed the MR diet for 6 h (supplementary Fig. S1). Activation of eIF2α by phosphorylation is the canonical path to ATF4 activation, but it was reported earlier that hepatic eIF2α phosphorylation was actually decreased after consuming the MR diet for 6 h^27^. An alternative mechanism of regulating ATF4 target genes has been described by Park et al.^30^, who showed that the mTOR inhibitor, Torin, inhibited ATF4-sensitive genes through a mechanism that was independent of eIF2α phosphorylation and involved mTOR-dependent stabilization of ATF4 by eIF4E-BP1. Notably, both hepatic ATF4 and eIF4E-BP1 are acutely increased by MR at the 6 h time point (Fig. 3c). To assess the involvement of mTOR in the acute ATF4-dependent induction of genes by MR, we treated HepG2 cells with Torin to inhibit mTOR and examined the ability of MR to induce our set of ISR and ATF4 target genes. As shown in Fig. 3e, Torin significantly reduced the induction of CHAC1, DDIT4, and FGF21 by MR, and completely blunted the induction of MARS, EIF4EBP1, PSAT1, SESN2, CARS and ASNS by MR. In addition, Torin significantly reduced basal expression of most of the genes in control cells (Fig. 3e). In contrast, the induction of SLC7A5 expression by MR was unaffected by mTOR inhibition (Fig. 3e). Collectively, these findings show that knockdown of ATF4 or inhibition of mTOR are effective in compromising the ability of MR to induce many of the genes involved in the ISR program.

**Figure 4:**
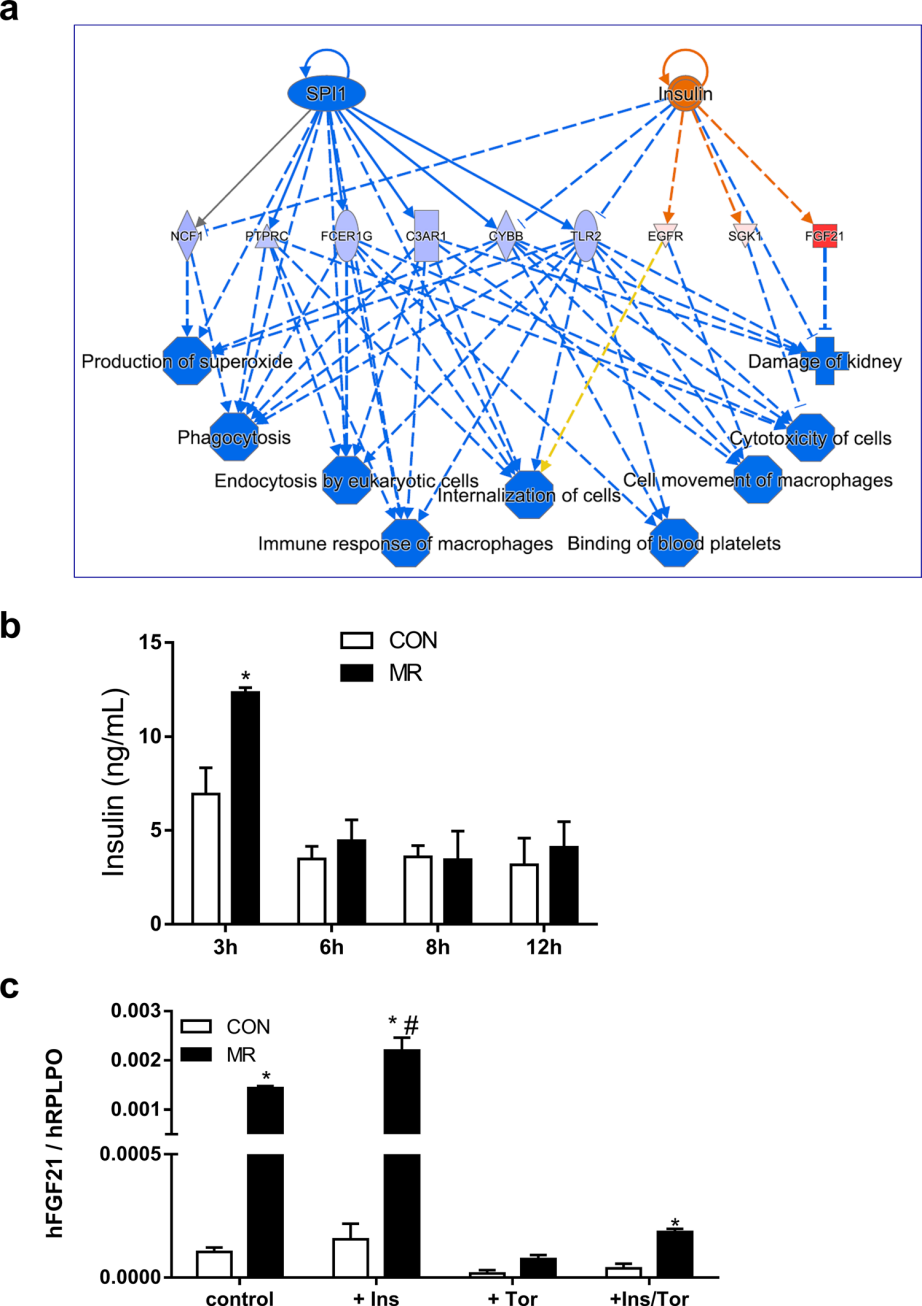
Activation of FGF21 by insulin is predicted by IPA (IPA Build version 458397 M; QIAGEN Inc., https://www.qiagenbioinformatics.com/products/ingenuity-pathway-analysis) after 6 h on MR (**a**). Serum insulin was measured from mice fed either Con or MR for 3 h, 6 h, 9 h, and 12 h (**b**). FGF21 expression was measured in HepG2 cells exposed to insulin (2 nM) in the presence and absence of Torin (200 nM) and cultured in either Con or MR (**c**). * Con different from MR; # MR without different from MR with 2 nM insulin; p < 0.05.

Next, we examined the signaling processes that may activate mTOR while general translation is coincidently repressed by limited methionine. Growth factors can activate mTOR through either Erk or Akt phosphorylation and subsequent inhibitory phosphorylation of Tuberous Sclerosis Complex 2 (TSC2)^42,43^. The early induction of FGF21 expression in HepG2 cells is mTOR dependent (Fig. 3e) and insulin was identified as a potential regulator of hepatic FGF21 through gene set enrichment analysis at the 6 h time point (Fig. 4a). Interestingly, in *Experiment 1* serum insulin was also temporarily increased in mice after 3 h of MR but at no other subsequent time point (Fig. 4b). To test for a link between insulin and mTOR-dependent effects on FGF21, HepG2 cells were treated with an insulin concentration similar to the one detected in serum of mice after 3 h MR (e.g., 2 nM). Insulin had no discernible effect on FGF21 mRNA in control cells and only modestly enhanced the MR-induced increase in FGF21 mRNA expression (Fig. 4c). As noted in Fig. 3e, Torin significantly reduced basal FGF21 expression in control cells (Fig. 4c), but it did not block the ability of insulin to potentiate the MR-induced increase in FGF21 expression (Fig. 4c). This finding indicates that insulin does not act upstream to activate mTOR but rather in parallel. Insulin also significantly increased the MR effect on CHAC1 and SLC7A5 expression but had no effect on other ATF4-responsive genes shown to be sensitive to mTOR (e.g., PSAT1, ASNS, CARS, MARS, SESN2, EIF4EBP1, and DDIT4 (data not shown)). Insulin, but not MR, increased TRB3 expression (data not shown).

**Figure 5:**
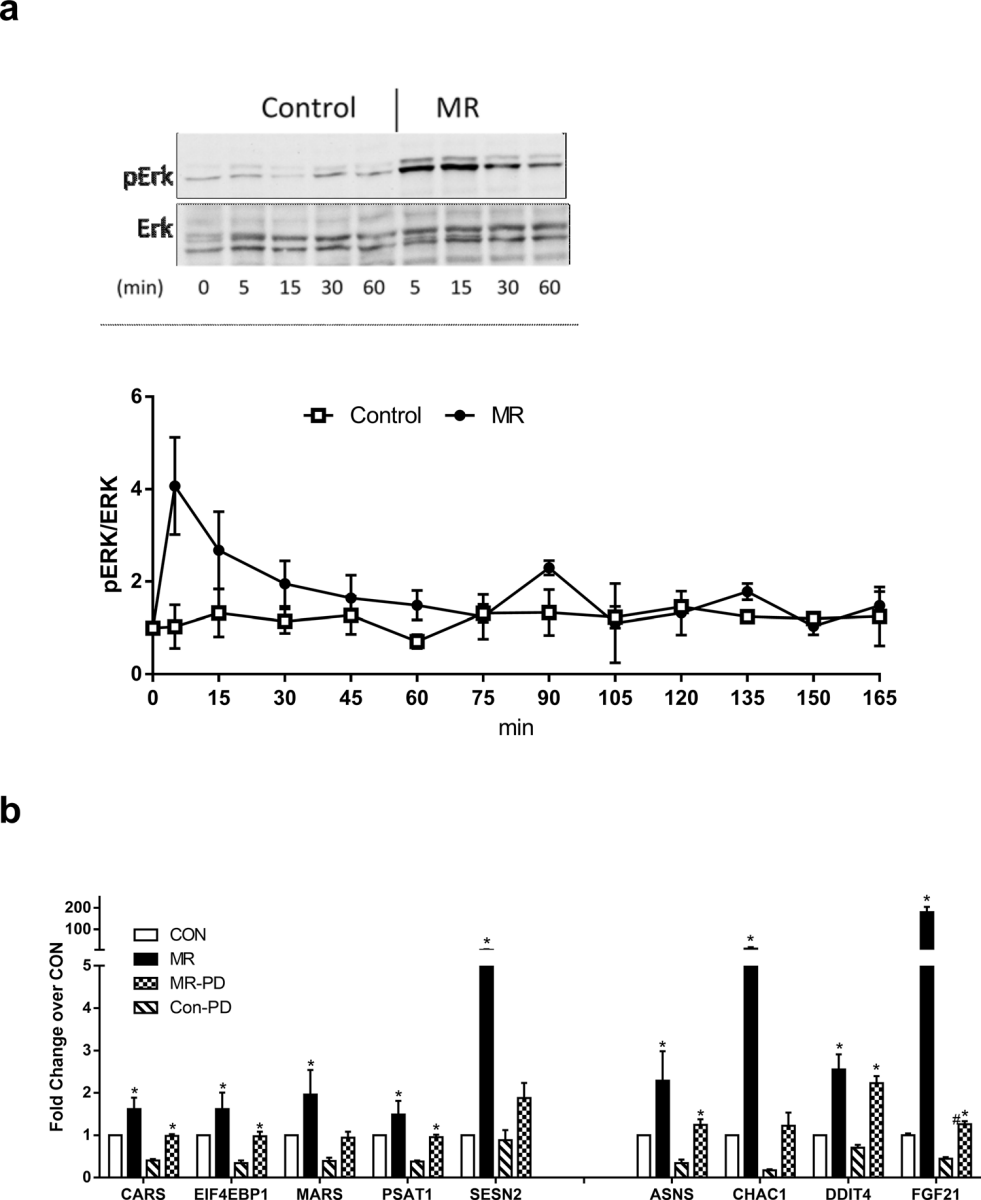
(**a**) HepG2 cells were exposed to either Con or MR media for 165 min and samples were taken after 5 min, and thereafter in 15 min intervals. Western blot analysis shows a representative detection of pErk and Erk up to 60 min exposure. Images are cropped, the full-length blot is presented in Supplementary Figure S2. The graph shows the average of pErk/Erk densitometric ratios from 5 independent experiments. (**b**) HepG2 cells were treated with Erk inhibitor PD98059 (50 µM) or vehicle and gene expression was measured after cultivation for 6 h in either Con or MR media. Data for HepG2 cells were obtained from at least two independent experiments with duplicates and fold induction was averaged. Data were compared between Con and MR for each gene using t-test and are presented as fold induction from Con. * Con different from MR; ^#^ MR without different from MR with PD98059; p < 0.05.

Regulatory input to mTOR can also be provided by Erk1/2, and analysis of canonical pathways acutely affected by MR detected a significant activation of hepatic Erk5 at the 6 h time point in mice (Fig. 3b). Erk5 is downstream of Erk1/2 but both are regulated by growth factors through activation of upstream Ras. To assess the possibility that MR could be signaling through Erk1/2 to regulate genes of the ISR, we compared phosphorylation of Erk1/2 in HepG2 cells after changing to fresh media containing either control or restricted levels of methionine. Erk1/2 phosphorylation was low and didn’t change over time in Con cells, while cells switched to the MR media showed a rapid increase in Erk1/2 phosphorylation within 5 min that quickly returned to control levels within 45 min and remained stable thereafter (Fig. 5a). To test the significance of MR-dependent activation of Erk1/2 to the MR-dependent induction of ISR genes, we inhibited Erk1/2 with PD 98059 (PD). Inhibition of Erk1/2 lowered basal expression of 7 of the 8 target genes and significantly blunted induction of some but not all of the genes by MR (Fig. 5b). The responses of the target genes fell into 3 main categories. For example, basal and MR-induced expression of DDIT4 was unaffected by ERK1/2 inhibition, while CARS, EIF4EBP1, PSAT1, and ASNS fell into a second category where PD reduced basal expression of each gene. However, MR produced the same fold-increase of each of these genes as it did in cells without the Erk1/2 inhibitor. The last category included MARS, SESN2, CHAC1, and FGF21, and their basal expression was also reduced by Erk1/2 inhibition. They differed from category 2 in that PD inhibited MR-induced induction of these genes to a far greater extent than the other genes (Fig. 5c). Collectively, these findings suggest that MR-dependent activation of Erk1/2 may provide another pathway to regulate the induction of ISR genes by MR. However, because of the differential effects of Torin and PD on basal expression of ISR genes and their differential effects on induction of ISR genes by MR, it seems unlikely that MR is signaling through Erk1/2 to regulate mTOR-induced effects on ISR genes. It seems more likely that the two signaling systems are providing independent but perhaps redundant input as mediators of the acute effects of MR on hepatic gene expression.

## Discussion

The liver functions as a sentinel to detect and respond to changes in dietary composition, and upon initiation of dietary MR, it quickly mobilizes reprogramming of the transcriptome and proteome in an evolutionarily conserved homeostatic response. The response is broadly referred to as the ISR^44^, and a key component of this early response is hepatic FGF21^45^. The corresponding increase in circulating FGF21 occurs within 6 h of starting the MR diet^5^, and over the next 4–5 days, the elevated FGF21 produces a coordinated increase in energy intake and energy expenditure that impacts energy balance. As shown here, the ISR also includes transcriptional responses that facilitate adaptation to the acute methionine insufficiency through rapid changes in RNA biology, including increased tRNA aminoacylation, decreased ternary complex formation, decreased peptide chain elongation, and a decrease in overall translation. These adaptive responses increase hepatic levels of amino acids, while activating PPARα mediated lipid metabolism and increasing carbohydrate metabolism. Despite the fivefold decrease in methionine intake, these compensatory adaptations limit the immediate decrease in hepatic methionine to twofold and even this decrease is mitigated within 2–4 days of starting the diet. The decrease in hepatic glutathione is also limited to ~ 50%, but this decrease seems to persist for as long as the MR diet is consumed. Together, these initial compensatory responses maintain redox homeostasis and precede the longer term metabolic adaptations that result in reduced adiposity, reduced tissue and circulating lipid levels, and an overall improvement in insulin sensitivity and metabolic health^3,5^. The longer term responses to MR have been extensively documented over the last decade^1,2,8,32,36,46–51^, and loss of function approaches have been used to assess the role of various sensing/signaling molecules, enzymes, and transcription factors in mediating the biological effects of dietary MR. However, the focus of much of this work has been to determine whether specific molecules such as GCN2^27^, PERK^24^, β_3_-adrenergic receptor^32^, leptin^52^, UCP1^49^, SCD1^53^, or FGF21^6^ are essential to specific components of the long term transcriptional, behavioral, and physiological responses to MR. It has been established that transcriptional activation of hepatic FGF21 is essential to many but not all of MR’s beneficial effects^6^.

In contrast, we have a far poorer understanding of how the liver senses the acute decrease in methionine and activates the ISR in the hours after initiating dietary MR. In the case of the essential amino acid sensor, GCN2, we predicted it would play a key role in detecting the immediate decrease in methionine and mediating the subsequent responses, but its absence failed to prevent either the acute transcriptional increase in hepatic *Fgf21* and other ATF4 target genes (supplementary Fig. S1) or longer term metabolic responses to the diet^27^. These findings suggest the existence of auxiliary systems capable of sensing and responding to decreased methionine intake. Similar findings were observed in liver-specific ATF4-null mice, where the acute induction of *Fgf21* by MR was dampened but not its induction after 10 weeks on the MR diet^54^. These findings argue that while ATF4 is normally important, other transcription factors can readily substitute for ATF4 in linking the reductions in methionine to adaptive transcriptional responses. An important remaining challenge is to identify the component molecules of these alternative sensing systems.

In the present work, we focused on the acute hepatic transcriptional and metabolomics profiles of mice in the 3–6 h time frame after introduction of dietary MR. The rationale for this approach comes from the observation that FGF21 is one target of a multi-component transcriptional program activated between 3 and 6 h after initiating the diet. Our strategy involved rigorous bioinformatic analysis of transcriptional changes within this time frame to make inferences about the signals and transcription factors known to regulate the affected biological processes. For example, the rapid decrease in hepatic methionine that occurs within 3 h is fully consistent with the predicted decrease in ternary complex formation (Fig. 2a,b, Table 1) caused by the precipitous shortage of methionine. This mechanism primarily impairs start site recognition due to insufficient Met-tRNA^28,29^. The bioinformatic analysis of RNAseq data also shows that general translation is inhibited at the level of ternary complex formation by limiting formation of initiator methionine tRNA. The net effect of these changes is to reduce consumption of amino acids, and this effect translated into the observed increase in hepatic levels of many essential and nonessential amino acids by 6 h. Upstream regulator analysis of the genes induced between 3 and 6 h and after 10 weeks were consistent with activation of ATF4 and NFE2L2 at the 6 h and 10 week time points. These findings are consistent with previous work proposing that MR activates the ISR and anti-oxidant response program in liver via a non-canonical PERK/NFE2L2 pathway that effectively senses the drop in methionine and its metabolites in the absence of GCN2^27^. GSH serves an essential role in disulfide bond formation during protein folding in the endoplasmic reticulum (ER), and ER stress is an established activator of PERK^55^. However, despite producing robust activation of PERK in liver, we found no evidence that MR increased ER stress or induced any components of the unfolded protein response^27^. Moreover, in subsequent work we found that the absence of PERK did not compromise the ability of MR to increase FGF21^24^. In contrast, we found that adding back cysteine to the MR diet reversed the chronic reduction of hepatic GSH by the diet and blocked its induction of FGF21^27^. We have suggested that the MR diet may be signaling to NFE2L2 through GSH, but the role of NFE2L2 as an essential mediator of the acute responses to MR remains an open question. The presence of hepatic NFE2L2 seems not to be necessary for either the acute or long-term induction of FGF21 by MR (authors’ unpublished data). Collectively, these findings argue that multiple alternative pathways can be recruited to mobilize the ISR during nutritional stress.

Analysis of RNAseq data identified changes in gene expression consistent with activation of ERK5 signaling at the 6 h time point. These findings raise the interesting possibility that MR may be using mTOR and/or ERK to provide transient signaling input from the evolutionarily conserved protein synthetic apparatus to transcriptional regulation of the ISR. mTOR also plays a key role in regulating amino acid transporters and tRNA synthetases by stabilizing ATF4 mRNA and enhancing its translation^30^. Thus with respect to methionine metabolism, there are several potential mechanisms that allow mTOR to function as a sensor and transcriptional mediator of dietary MR.

To test the role of these signaling systems, along with ATF4 during the acute activation of the ISR in hepatocytes, we evaluated short term changes in ten representative ISR genes after restriction of media methionine in an in vitro model of MR in HepG2 cells. We used knockdown of ATF4, inhibition of mTOR with Torin, or inhibition of ERK with PD98059 to test the hypotheses that MR-dependent stress signaling was communicating with ATF4 through mTOR and/or ERK. Knockdown of ATF4 demonstrated the importance of ATF4 in this model system by blunting or blocking the MR-dependent induction of all but one of the ten genes. Torin significantly reduced the induction of CHAC1, DDIT4, and FGF21 by MR, and completely blunted the induction of MARS, EIF4EBP1, PSAT1, SESN2, CARS and ASNS by MR. In addition, Torin significantly reduced basal expression of most of the genes in control cells. Park et al.^30^ made similar observations in HEK293 cells where Torin inhibited expression of several ATF4 target genes. The affected genes include tRNA synthetases, amino acid transporters, SESN2, CHAC1, and DDIT4; a group of genes similar to the group induced by MR in HepG2 cells and in mouse liver after 6 h of MR. Park’s data shows that Torin inhibits ATF4 translation by destabilizing its mRNA^30^, whereas activation of mTOR induces ATF4 target genes by stabilizing ATF4 mRNA. The MR-dependent mTOR activation would effectively increase ISR genes by increasing ATF4 translation. Further studies will be needed to confirm this possibility and establish how changes in ternary complex formation may be linked to mTOR activity. Canonical mTor pathways activate translation through phosphorylation of S6 kinase and eIF4E-BP1, whereas MR inhibits translation. We did not detect any changes in mTor, S6, S6k and eIF4E-BP1 phosphorylation in either mouse liver extracts after 1, 3, and 6 h on MR or in HepG2 cells up to 165 min in MR media (author’s unpublished data). Therefore, MR must be acting through non-canonical Torin-sensitive mTor pathways. Irrespective of mechanism, our in vitro data make a strong case that inhibition of mTOR activity impairs MR-dependent activation of the ISR.

To examine the potential role of Erk1/2 as an MR-dependent stress signal linked to the ISR in HepG2 cells, we tested whether MR affected Erk1/2 activity and whether inhibition of Erk1/2 impaired MR-dependent induction of ISR genes. MR produced a rapid but transient activation of Erk1/2 and its inhibition lowered basal expression of 7 of the 9 target genes. Erk1/2 inhibition also significantly blunted the MR-induced increases of most ISR genes by MR. Collectively, these findings suggest that MR-dependent activation of Erk1/2 may provide another pathway to regulate the induction of ISR genes by MR. However, because of the differential effects of mTOR and Erk1/2 inhibition on basal expression of ISR genes and the differential effects of the inhibitors on which genes were induced by MR, it seems unlikely that MR is signaling through Erk1/2 to regulate mTOR-induced effects on ISR genes. It seems more likely that the two signaling systems are providing independent but perhaps redundant input as mediators of the acute effects of MR on hepatic gene expression. We also found no evidence that insulin was providing signaling input to mTOR or Erk1/2 in their regulation of ISR genes.

Collectively, our studies provide compelling new evidence that mTOR and Erk1/2 are being acutely regulated by dietary MR to provide regulatory input to the hepatic ISR in the hours after initiation of the diet. Our findings suggest that the acute inhibition of ternary complex formation by the shortage of methionine has an immediate, profound effect on RNA biology to limit protein translation so it seems possible that these responses may represent the initial stress signaling mechanism. It remains to be established how these transient changes within the hepatocyte activate mTOR and Erk1/2 to provide regulatory input to the ISR.

## Methods

### Animals, diets, and cell lines

All experiments were reviewed and approved by the Pennington Institutional Animal Care and Use Committee using guidelines established by the National Research Council, the Animal Welfare Act, and the Public Health Service Policy on the humane care and use of animals. Pelleted diets (Dyets Inc., Bethlehem, Pennsylvania) containing either 0.86% (Con) or 0.17% methionine (MR) were provided ad libitum to the mice in each experiment as before^32^. The diets were isocaloric at 15.96 kJ/g, with 18.9% of energy from fat (corn oil), 64.9% from carbohydrate, and 14.8% from a custom mixture of L-amino acids. The details of amino acid content are provided in Table 2. Temperature was maintained at 22–23 °C. Two experiments were conducted to examine the acute and chronic responses to dietary MR.

*Experiment 1—*This experiment was designed to examine the acute responses to dietary MR over the first 8 days after introduction of the diet. Male C57BL/6 J mice were obtained from Jackson Labs (Bar Harbor, ME, USA) at 7 weeks of age and adapted to the Control (Con) diet and a reverse light cycle for a period of 10 days with the lights on between 8 pm and 8 am, and lights off between 8 am and 8 pm. During this period, food was removed every night at 8 PM and restored at 8 AM. After the 10 days of adaptation, the mice were randomized into fourteen groups of 8 mice (body weight was 23.2 ± 1.2 g). At 8 AM on the 11th day (e.g., Time 0), seven groups continued receiving the Con diet while seven groups were switched to the MR diet. From this time 0 forward, one group of Con mice and one group of MR mice were euthanized exactly 3 h, 6 h, 9 h, 12 h, 2 days, 4 days, and 8 days thereafter. Experiments with mice on the diets for 2 days, 4 days, and 8 days ended in the middle of the dark cycle. At each time point, mice were exposed to CO_2_, decapitated, and trunk blood was harvested. Livers were removed and snap frozen in liquid nitrogen. Serum was obtained from clotted blood after centrifugation and stored at − 80 °C.

*Experiment 2—*Eight week old male C57BL/6 J mice were adapted to Con diet for 10 days. Thereafter, 16 mice were randomized into two groups of 8 mice and fed either the Con or MR diet for 10 weeks. The mice were then euthanized and tissues harvested as before. The studies were carried out in compliance with the ARRIVE guidelines (http://www.nc3rs.org.uk/page.asp?id=1357).

In vitro* HepG2 experiments*—HepG2 cells were exposed to DMEM media containing Con (0.2 mM) or MR (0.01 mM) levels of methionine and cysteine as described before^5^ for various time periods. In some experiments, cells were pre-treated for 30–60 min with inhibitors for either Erk (PD 98059; 50 µM; Sigma-Aldrich Corp. St. Louis, MO) or mTOR (Torin; 200 nM; ThermoFisher Scientific, Pittsburgh, PA). The role of ATF4 in HepG2 cells was assessed by reverse transfection using RNAiMax (ThermoFisher Scientific, Pittsburgh, PA) according to manufacturer’s instructions. HepG2 cells were plated onto the transfection mix containing either control or ATF4 siRNA (Santa Cruz Biotechnologies, Dallas, TX), and 24–48 h later, cells were cultured in either Con or MR media for 6 h. Erk phosphorylation was assessed in 50 µg HepG2 extracts run on 10% gels and transferred onto a PDVF membrane. Membranes were blotted with a well-established antibody against phosphorylated Erk (#9101; Cell Signaling; Danvers, MA), stripped and blotted with an antibody against Erk (#9102; Cell Signaling; Danvers, MA).

### Metabolomics analysis

Frozen liver (30–50 mg) was powdered in liquid nitrogen and submitted to the Mass Spectrometry Core at UT Knoxville for non-targeted metabolomics as described in^33^. Data were normalized to sample weight and relative quantities of amino acids were compared between Con and MR fed mice.

Hepatic amino acid content was also analyzed at the DUKE-NUS Metabolomic Core Facility (Singapore). Methods were modified from Newgard^34^ and Sinha^35^. Absolute quantitation of amino acids was done by comparing the ratios of the metabolites to their respective internal standards, and against an external calibration curve.

### RNA isolation and RNAseq

Total RNA was isolated from HepG2 cells and livers using RNeasy Mini kit (Qiagen, Valencia, CA). RNA concentrations were measured using Nanodrop ND-1000 spectrophotometer (Nanodrop Technologies, Wilmington, DE). Total RNA was analyzed using the Agilent Bioanalyzer RNA 1000 chip as a QC step to determine the quality of the RNA. Samples were verified to have RIN values of > 7, indicating high quality RNA. Samples were then processed for library construction using the Lexogen Quant-Seq 3′ mRNA-Seq Library Prep Kit. Completed libraries were analyzed on the Agilent Bioanalyzer High Sensitivity DNA chip to verify correct library size. All libraries were pooled in equimolar amounts and sequenced on the Illumina NextSeq 500 at 50 bp forward read and 6 bp forward index read. Primary data analysis was performed using the Lexogen Quantseq pipeline V1.8.8 on the Bluebee platform for quality control, mapping, and read count tables. The gene expression profiles were assessed from 6 replicates for each tissue for each dietary group. CLC Genomics Workbench was used to process data. The expression profiling data has been deposited in NCBI under GEO accession GSE158068.

Analysis of gene expression profiles from *Experiment 2* (e.g., 8 weeks of dietary MR) was described previously^36^. Data from that analysis were used to compare canonical pathways and upstream analysis with data from RNAseq of mouse livers after 6 h MR.

### Bioinformatics analysis

For both 3 h and 6 h RNAseq datasets, pathway enrichment analysis was conducted using the pre-rank option in GSEA (GSEAprerank)^37^. DESeq2-derived log fold-change estimates were used to rank genes. Genes with a maximum TPM >  = 5 were retained for GSEA analysis, resulting in 8407 genes for 3 h data and 8704 genes for 6 h data. Pathway enrichment was based on the Reactome pathway database obtained from MSigDB (https://www.gsea-msigdb.org/gsea/msigdb/index.jsp), with pathways containing >  = 15 or <  = 250 genes retained for enrichment analysis. The significantly enriched pathways from each time point were further compared based on their gene content similarity via EnrichmentMap^38^ using the following parameters: enrichment p-value cutoff = 0.005, similarity cutoff (overlap coefficient) >  = 0.5). Results from Enrichment Map analysis were visualized in Cytoscape^39^.

In addition to GSEA, pathway over-representation analysis was also conducted using Ingenuity Pathway analysis (IPA; QIAGEN Inc., https://www.qiagenbioinformatics.com/products/ingenuity-pathway-analysis), considering 274 differentially expressed genes from the 6 h data (absolute log fold-change >  = 1.3, FDR >  = 0.3). Within IPA, the Upstream Regulator Analysis module was utilized to identify putative gene regulators responsible for the observed transcriptional patterns at 6 h. Several possible types of regulators were considered including transcription factors, cytokines, enzymes, G-protein coupled receptors, protein kinases, ligand dependent nuclear receptors and translation regulators. Regulators with an activation z-score > 2 or < − 2 were considered to be activated or inhibited, respectively.

### Serum insulin

Serum insulin of mice exposed to MR for 3 h, 6 h, 9 h, and 12 h was analyzed using ELISA (Millipore; Billerica, MA) according to the manufacturer’s instructions.

### qPCR analysis

Quantitative PCR analysis of mouse liver and HepG2 mRNA expression was performed after reverse transcription of 2 µg RNA into cDNA. Gene expression was measured using 10 ng cDNA and SYBR (Biorad). Data were normalized to either cyclophilin (mouse liver) or 60S acidic ribosomal protein P0 RPLP0 (HepG2 cells) expression. Primer sequences are provided in supplementary Table S1.

### Statistical analysis

Data was analyzed using GraphPad Prism (GraphPad Software; San Diego, CA) and presented as mean ± standard error. Statistical significance was tested using a two-tailed Student t test, with protection against type I errors set at 5%.

## Supplementary Information

Supplementary Information.
