# Supplementary material for: The acute transcriptional responses to dietary methionine restriction are triggered by inhibition of ternary complex formation and linked to Erk1/2, mTOR, and ATF4
Source: Sci Rep. 2021 Feb 12;11:3765. doi: 10.1038/s41598-021-83380-0 (PMC7880992; doi:10.1038/s41598-021-83380-0)
Supplement: Supplementary file 1 — Supplementary Information. [file 41598_2021_83380_MOESM1_ESM.pdf]

# The acute transcriptional responses to dietary methionine restriction are triggered by inhibition of ternary complex formation and linked to Erk1/2, mTOR, and ATF4

Kirsten P Stone[1], Sujoy Ghosh[2,3], Jean Paul Kovalik[3], Manda Orgeron[1],
Desiree Wanders[4], Landon Sims[1], Thomas W Gettys[1]

[1]Laboratory of Nutrient Sensing & Adipocyte Signaling,
[2]Laboratory of Computational Biology
Pennington Biomedical Research Center, Baton Rouge, LA, USA

[3]Program in Cardiovascular and Metabolic Disorders and Center for Computational Biology,
Duke-NUS Medical School, Singapore

[4]Department of Nutrition, Georgia State University, Atlanta, GA, USA

**Supplementary Figure S1 pertaining to Figure 3D.**

GCN2 is not required for MR induced ATF4 target gene expression. Wild-type and Gcn2$^{-/-}$ mice were fed either Con or MR diet for 6h. Gene expression of Atf4 target genes was evaluated using qPCR. Experimental details are described in D. Wanders, et al., "Role of GCN2-independent signaling through a non-canonical PERK/NRF2 pathway in the physiological responses to dietary methionine restriction," Diabetes 65(6), 1499 (2016). *p<0.05 Con vs MR, #p<0.05 WT MR vs Gcn2$^{-/-}$ MR.

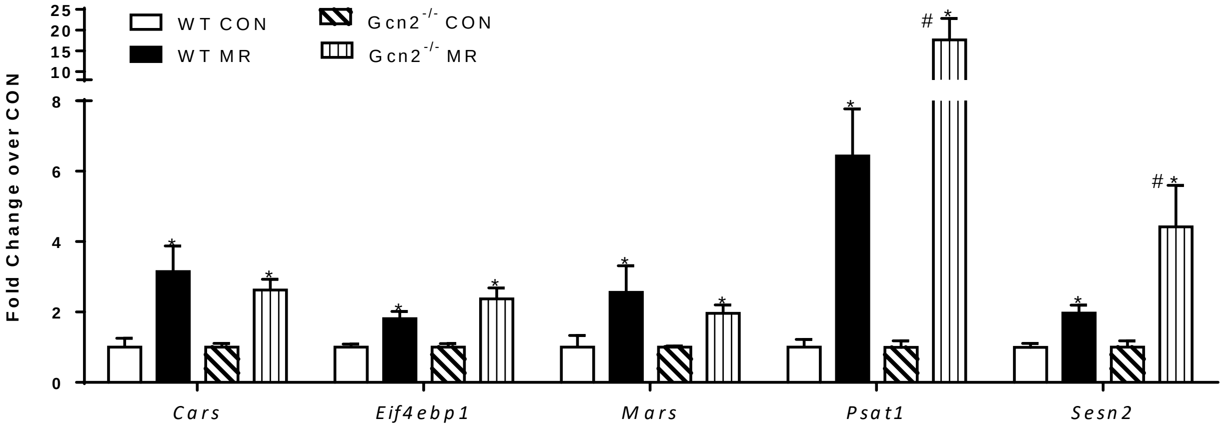

**Supplementary Figure S2 pertaining to Figure 5A**.

Images are from a blot loaded with 50µg of extracts from HepG2 cells exposed for the annotated time to Control media (CON), methionine restricted (MR), or leucine restricted (LR) media. The blot was first probed with Cell Signaling antibody against pErk (#9101), then secondary anti rabbit, and visualized using ECL for 30 seconds. The same membrane was stripped and incubated with anti Erk antibody (#9102) for normalization of the phospho signal. Both antibodies are widely used and cited in several thousands of publications. For creating Figure 5A, both images were cropped horizontally to leave on both sides approximately six band widths above and below the band. They were also cropped vertically to exclude the LR results.

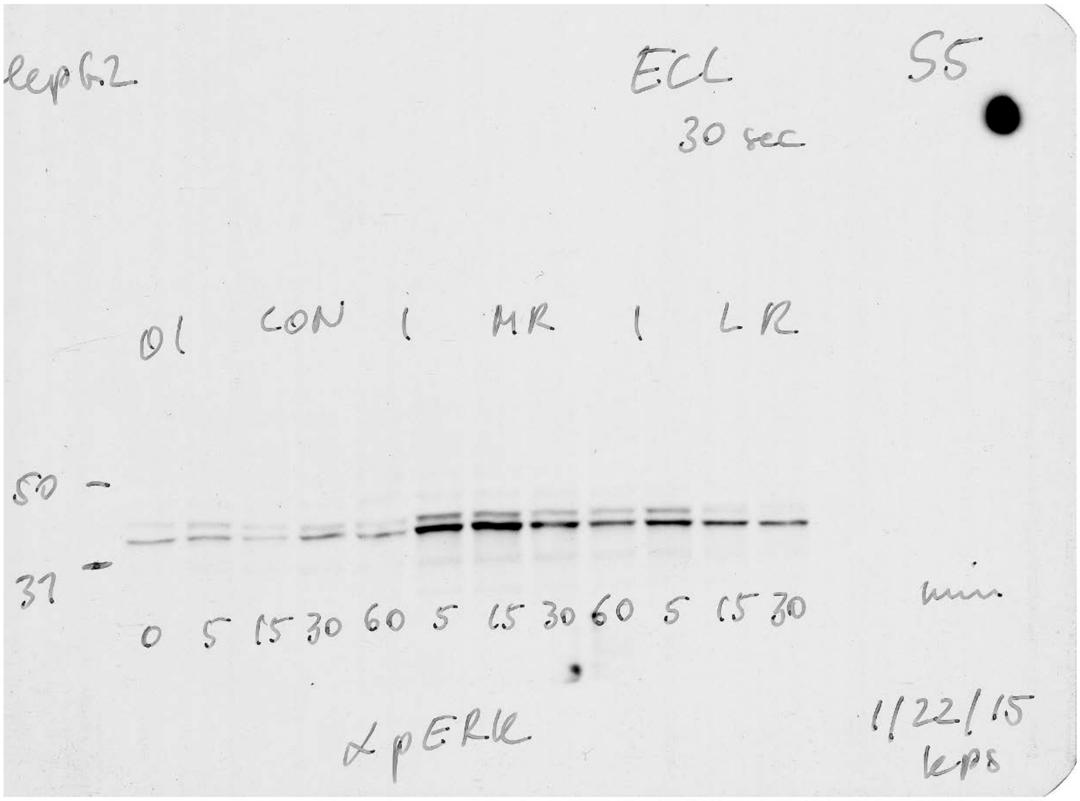
HepG2
ECL
30 sec
S5
01 CON | MR | LR
50 -
37 -
0 5 15 30 60 5 15 30 60 5 15 30
min
α pERK
1/22/15
kps

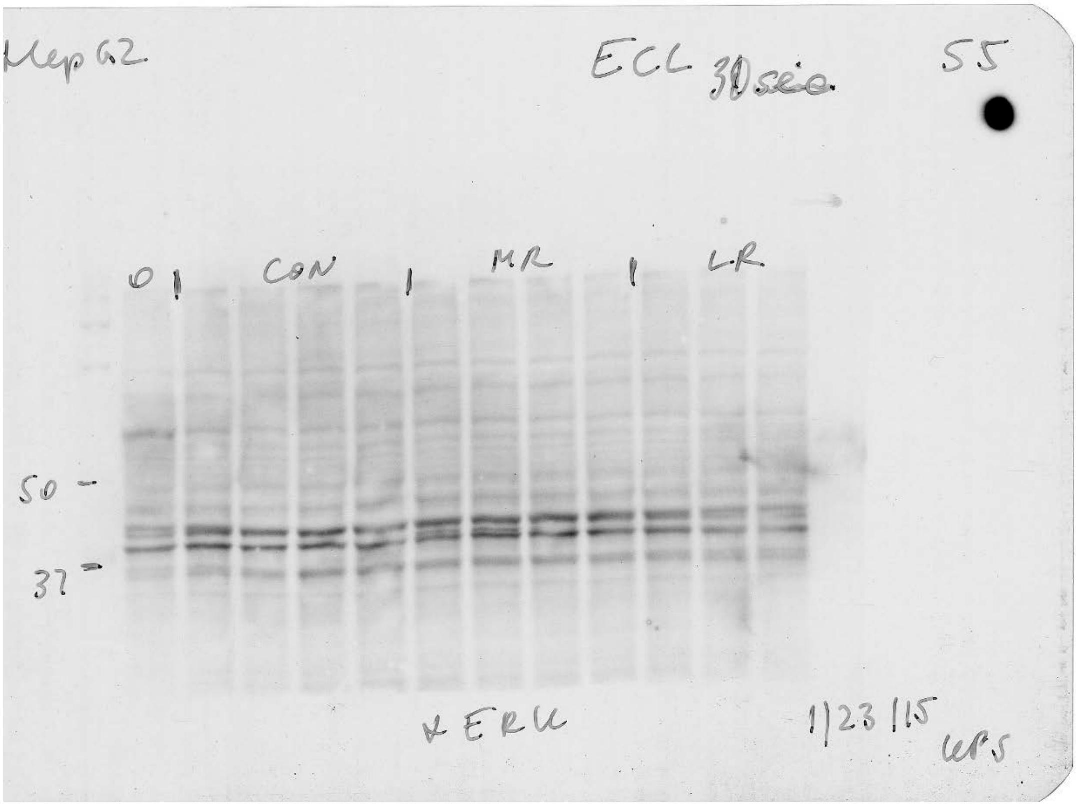
HepG2
ECL 30 sec
S5
0 | CON | MR | LR
50 -
37 -
α ERK
1/23/15
kps

**Supplemental Table S1.** Primer sequences for qPCR.

| Gene | Forward | Reverse |
|---|---|---|
| ***Mouse*** | | |
| *Asns* | GGGGGCCTGGACTCGAGCTT | TTGCCACCTTTCTAGCGGCCA |
| *Atf4* | GGAATGGCCGGCTATGG | TCCCGGAAAAGGCATCCT |
| *Cars* | GATGCGTCTCACATGGGACAT | CCCTCCGCAGGATATCGA |
| *Chac1* | TATAGTGACAGCCGTGTGGG | GCTCCCCTCGAACTTGGTAT |
| *Ddit4* | ACCGGCTTCAGAGTCATCAA | TCAGGTGGCTATCGTCAGTT |
| *Eif4ebp1* | GTTGCTGGAGGGTCGTGG | TCCCAGGTAACCCAGCCT |
| *Fgf21* | TGACACCCAGGATTTGAATGAC | GCAGCCAATGATGTGTGCTTAC |
| *Mars* | TACCATTCCTTACCCGGCCTA | GCAGATTGCACTAGCAGAGAA |
| *Psat1* | CAGTGGAGCGCCAGAATAGAA | CCTGTGCCCCTTCAAGGAG |
| *Sesn2* | GACCATGGCTACTCGCTGAT | GCTGCCTGGAACTTCTCATC |
| *Slc7a5* | TTGAAGGCACCAATCTGGACG | GGAGATGATGATGGCCAGGG |
| *Trb3* | CCCGGTGCCGCAGCACTTTA | GCTCGCATCTTGTCCTGGAGCC |
| *cyclophilin* | CTTCGAGCTGTTTGCAGACAAAGT | AGATGCCAGGACCTGTATGCT |
| | | |
| ***HUMAN*** | | |
| ASNS | GCAGCTGAAAGAAGCCCAAGT | TGTCTTCCATGCCAATTGCA |
| ATF4 | CCCTTCACCTTCTTACAACCTC | TGCCCAGCTCTAAACTAAAGGA |
| CARS | GCTCTTGGAGGATGTTCAGG | CTTCCTCTCCCGTGAGTCTG |
| CHAC1 | CCTGAAGTACCTGAATGTGCGAGA | GCAGCAAGTATTCAAGGTTGTGGC |
| DDIT4 | ACGAGAAGCGGTCCCAAAG | CACTCTGAGTTCATCAGCAAAGG |
| EIF4EBP1 | CTGATGGAGTGTCGGAAC | ATGGCTGGTGCTTTAAATG |
| FGF21 | GGGAGTCAAGACATCCAGGT | GGCTTCGGACTGGTAAACAT |
| MARS | GCGCTTCTATCTGCTGTACA | TAGACACAAACATCCCAGCT |
| PSAT1 | GCTTGGTTCTGGAGTGGATT | GTCCCACCAGCTTTACAG |
| SESN2 | TTCGGATATGAGGACTTC | ATGGTATTGTAGGTGAGG |
| SLC7A5 | GAAGGCACCAAACTGGATGT | GAAGTAGGCCAGGTTGGTCA |
| RPLP0 | TTCTCCTTTGCGCTGGTCAT | CAGGGAGCGAGAATGCAGAGT |
